# Supplementary material for: Splice modulators target PMS1 to reduce somatic expansion of the Huntington’s disease-associated CAG repeat
Source: Nat Commun. 2024 Apr 12;15:3182. doi: 10.1038/s41467-024-47485-0 (PMC11015039; doi:10.1038/s41467-024-47485-0)
Supplement: Supplementary file 1 — Supplementary Information [file 41467_2024_47485_MOESM1_ESM.pdf]

Contents

Supplementary Figure 1 2
Supplementary Figure 2 3
Supplementary Figure 3 4
Supplementary Figure 4 6
Supplementary Figure 5 7
Supplementary Figure 6 8
Supplementary Figure 7 9
Supplementary Figure 8 10
Supplementary Figure 9 11
Supplementary Figure 10 12
Supplementary Figure 11 13
Supplementary Figure 12 14
Supplementary Figure 13 15
Supplementary Table 1: primers 16
Supplementary Table 2: sgRNAs 17

Supplementary Figure 1

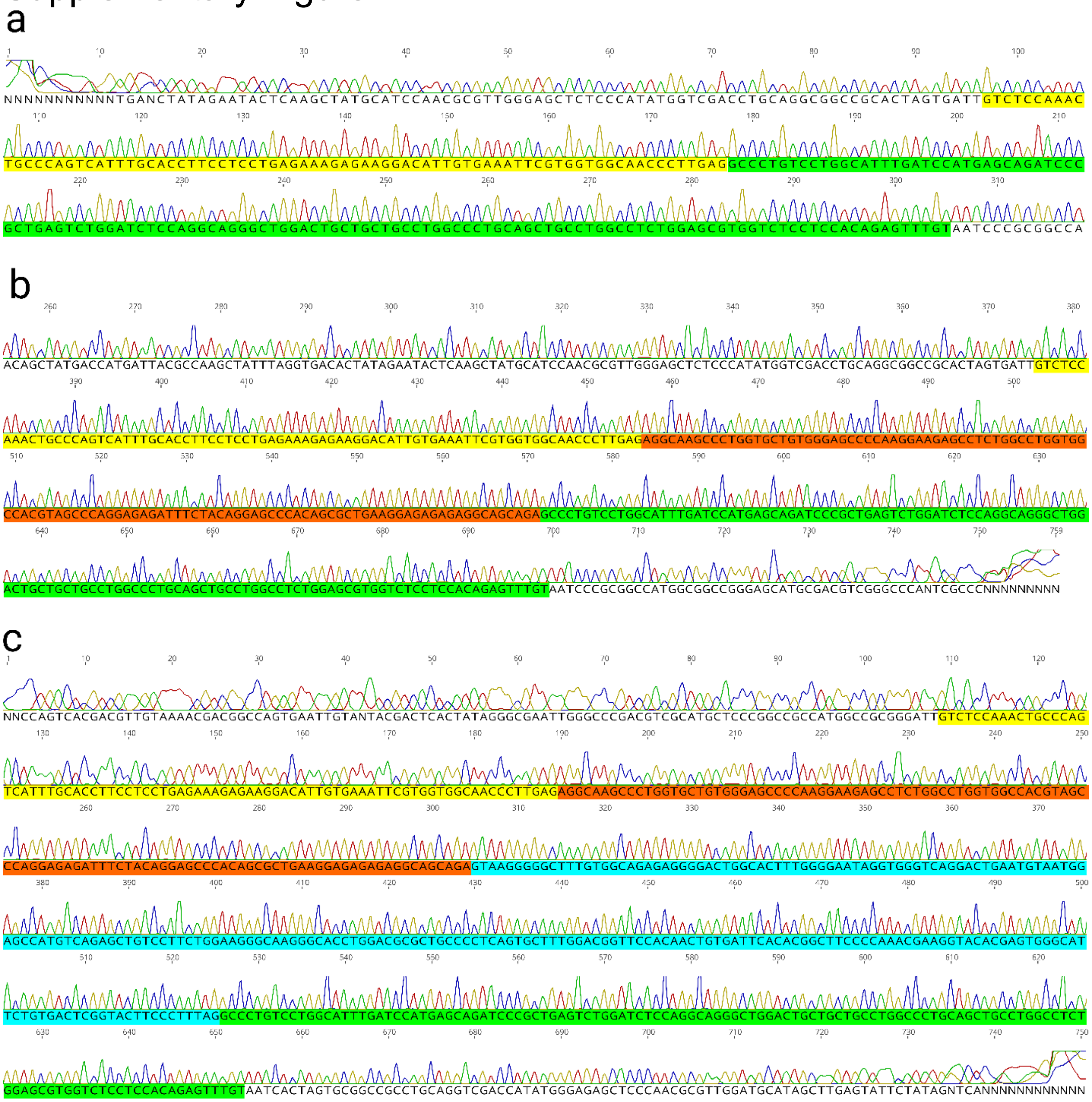

Supplementary Figure 1. Sanger sequencing for TA-cloned PCR fragments for: (a) *HTT* canonical exon 49-50 splicing, (b) pseudoexon inclusion exon 49-50a-50, (c) alternative splice site exon 49-50b. Yellow is exon 49 sequence, orange is exon 50a, blue is the entire intronic sequence between exon 50a and exon 50, green is exon 50.

Supplementary Figure 2

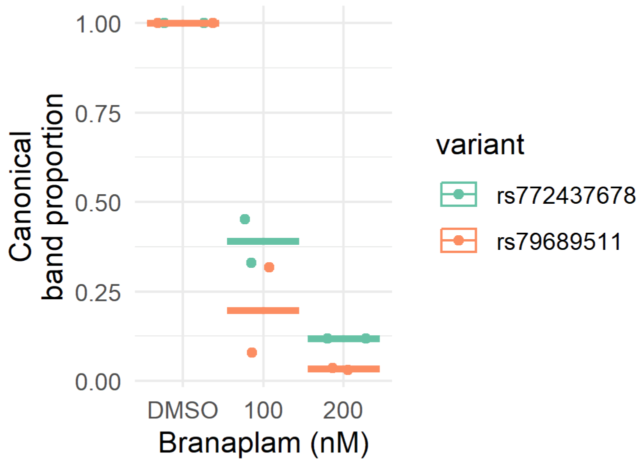

Supplementary Figure 2. Genetic variants and *HTT* splice modulation. Canonical *HTT* exon 49-50 for cell lines containing variants rs79689511 (orange) and rs772437678 (teal) for 100 and 200 nM branaplam. Source data are provided as a Source Data file.

Supplementary Figure 3

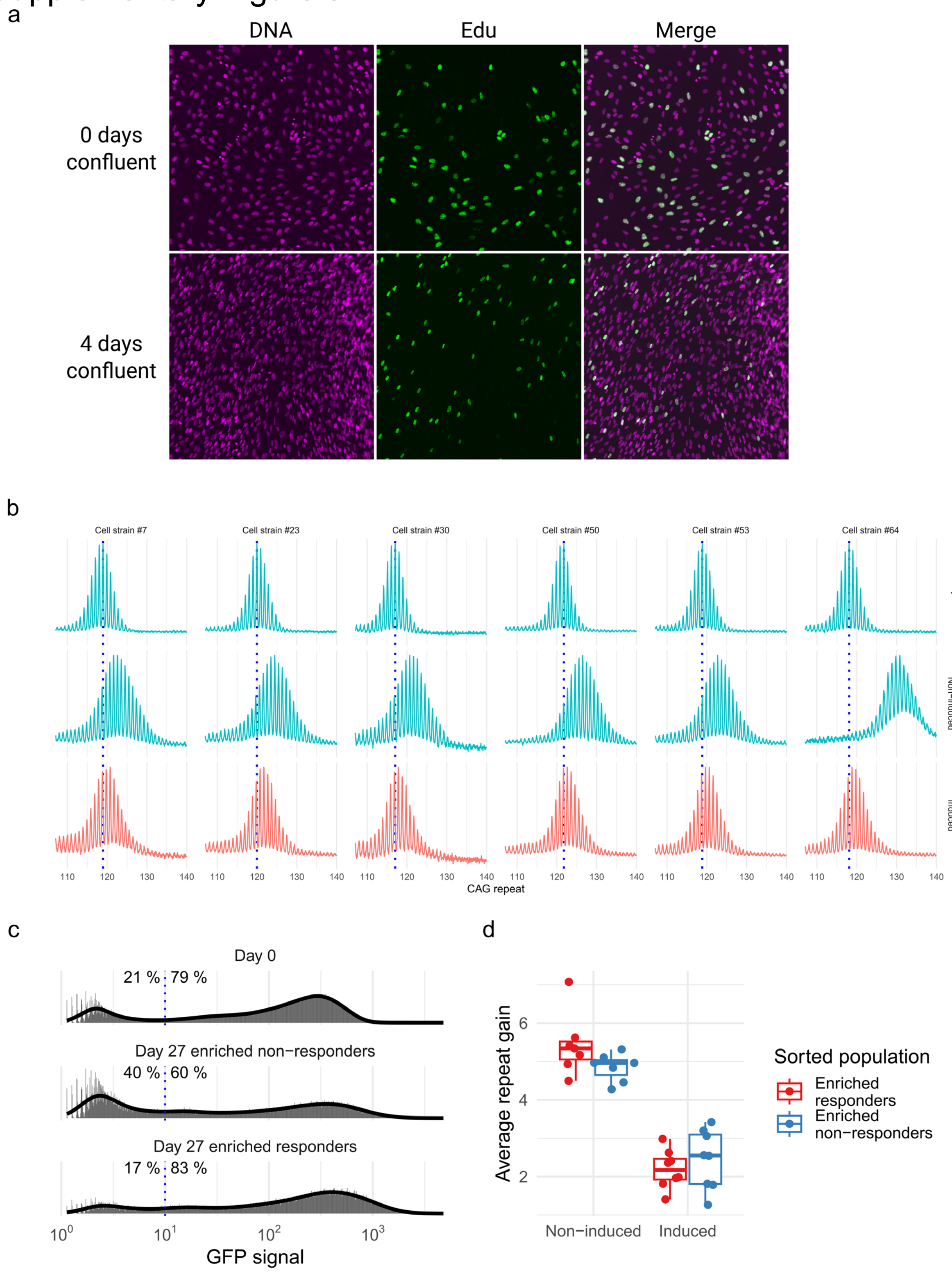

Supplementary Figure 3. RPE1-AAVS1-CAG115 cell cycle analysis, repeat traces, and instability of transgene doxycycline non-responders. (a) Representative images for S-phase image analysis, showing DNA and Edu stains for cultures confluent for 0 and 4 days. (b) CAG repeat traces of different clonal cell strains showing the change in repeat distribution from day 0 (top) for non-induced (middle, blue) and induced (bottom, red) at day 28. (c) GFP signal analyzed by flow cytometry at day 0 (top) which was used to separate GFP negative (non-responders) and GFP positive (responders) populations. These populations were then used for parallel repeat instability experiments. After 27 days, the initial non-responders (middle) and initial responders (bottom) were analyzed for GFP signal. The proportion of the population to the left (GFP-negative) or right (GFP-positive) of the dotted line is given for each population. (d) Average repeat gain for the initial responders (red) or initial non-responders (blue), compared to the sorted population of each at day 0, either non-induced or induced with doxycycline. For each group 7-8 cultures were analyzed. Box plots show the 25th and 75th percentiles (box), median (horizontal line), and range (whiskers, which are capped at 1.5x the inter-quartile range). Source data are provided as a Source Data file.

Supplementary Figure 4

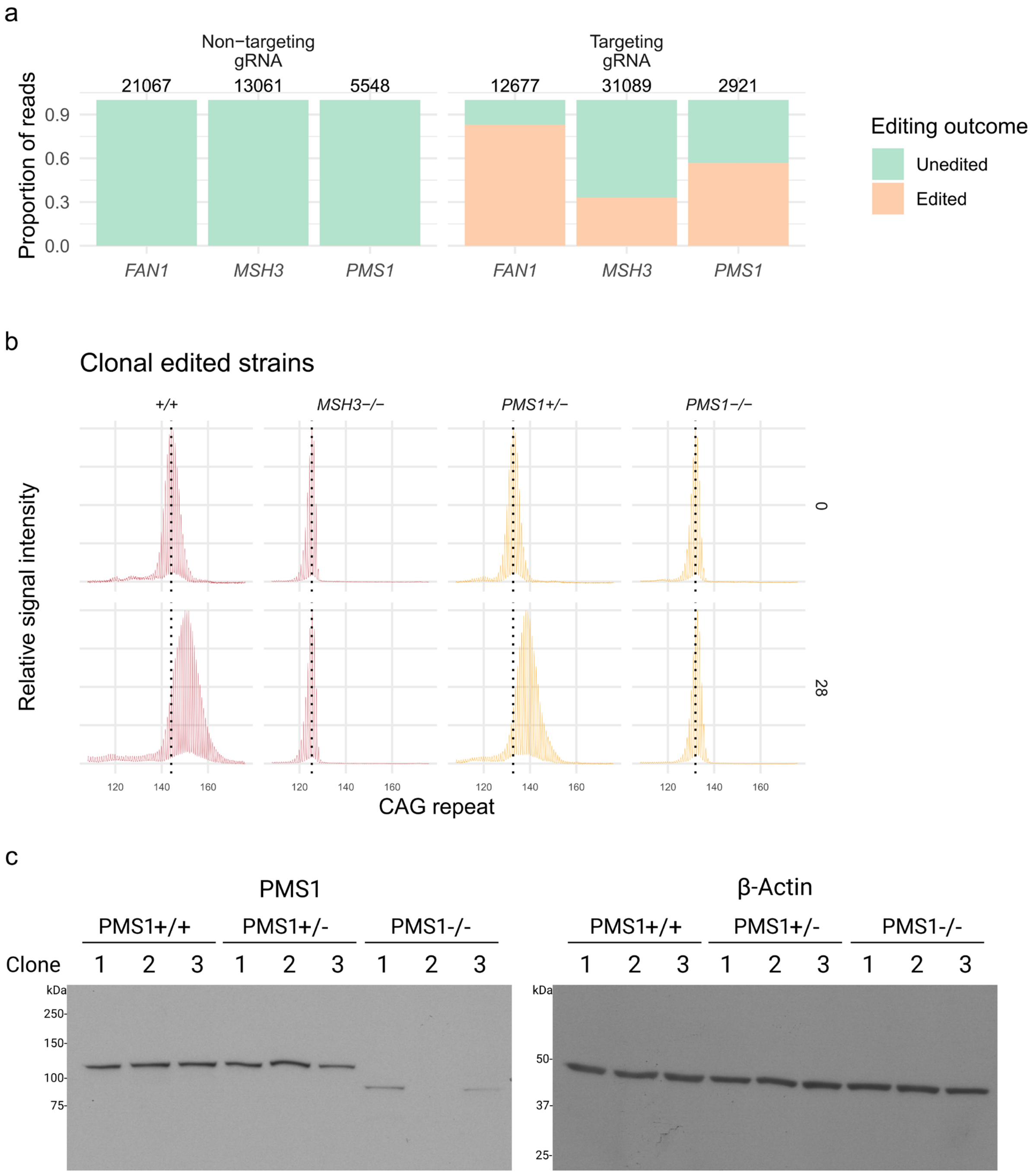

Supplementary Figure 4. RPE1-AAVS1-CAG115 HD modifier genome editing. (a) Proportion of edited reads in samples transfected with either non targeting or targeting gRNAs in RPE1-AAVS1-CAG115. The numbers above the bars indicate the total reads analyzed. (b) Representative fragment analysis traces of isolated clonal edited strains from either *MSH3* (red) or *PMS1* (orange) targeted populations. (c) Western blot for PMS1 and beta-Actin for the *PMS1* non-edited cell clones (PMS1+/+), heterozygously edited (PMS1+/-) and homozygously edited (PMS1-/-). The samples are from a single experiment with three biologically independent cell clones per genotype. Source data are provided as a Source Data file .

Supplementary Figure 5

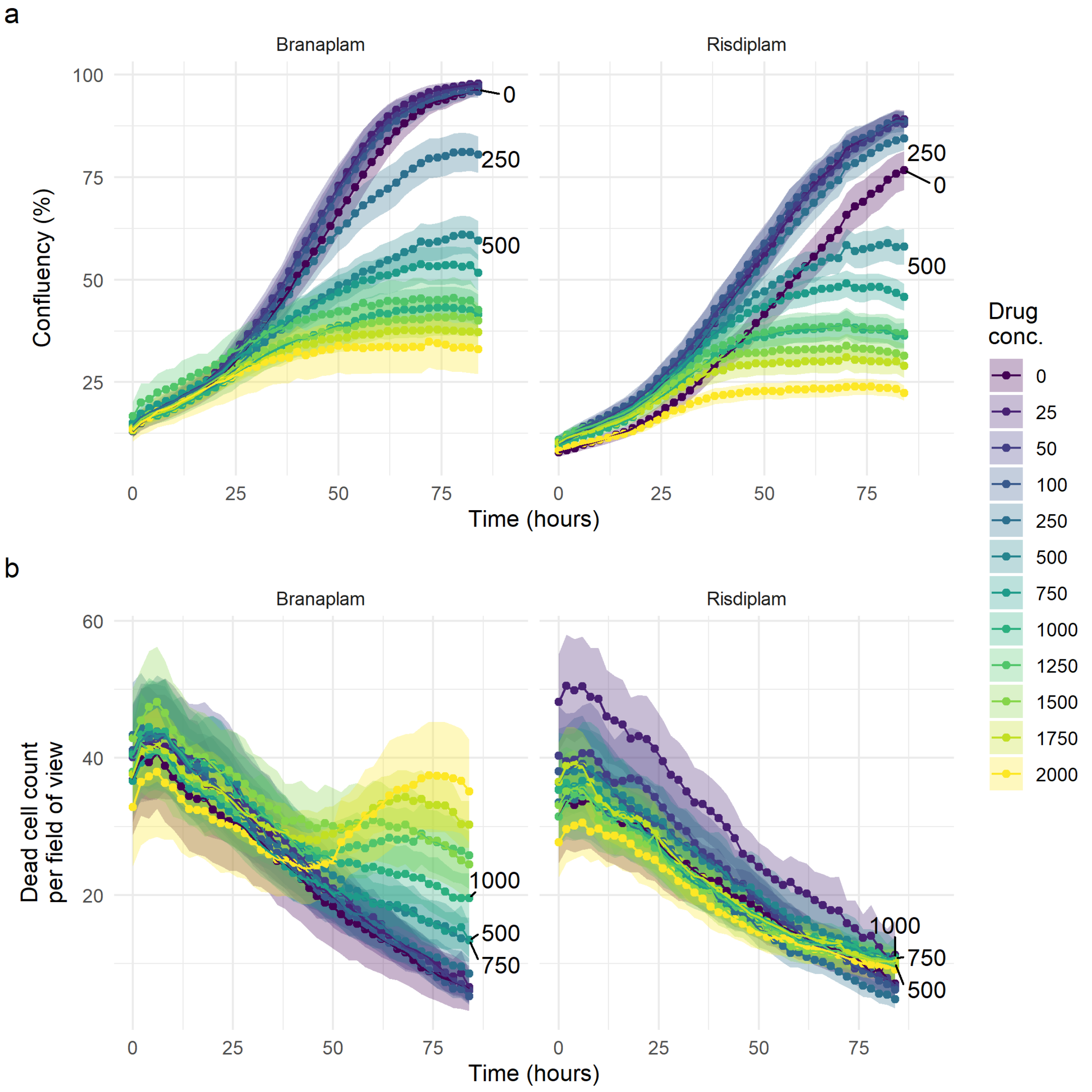

Supplementary Figure 5. The effect of branaplam and risdiplam on cell growth and acute cytotoxicity. (a) High-throughput image analysis for quantification of confluency over time with the treatment of branaplam (left) or risdiplam (right). The drug concentration is represented by the color, with key concentrations labelled on the plot. (b) Quantification of DNA labelling of dead cells. Source data are provided as a Source Data file.

Supplementary Figure 6

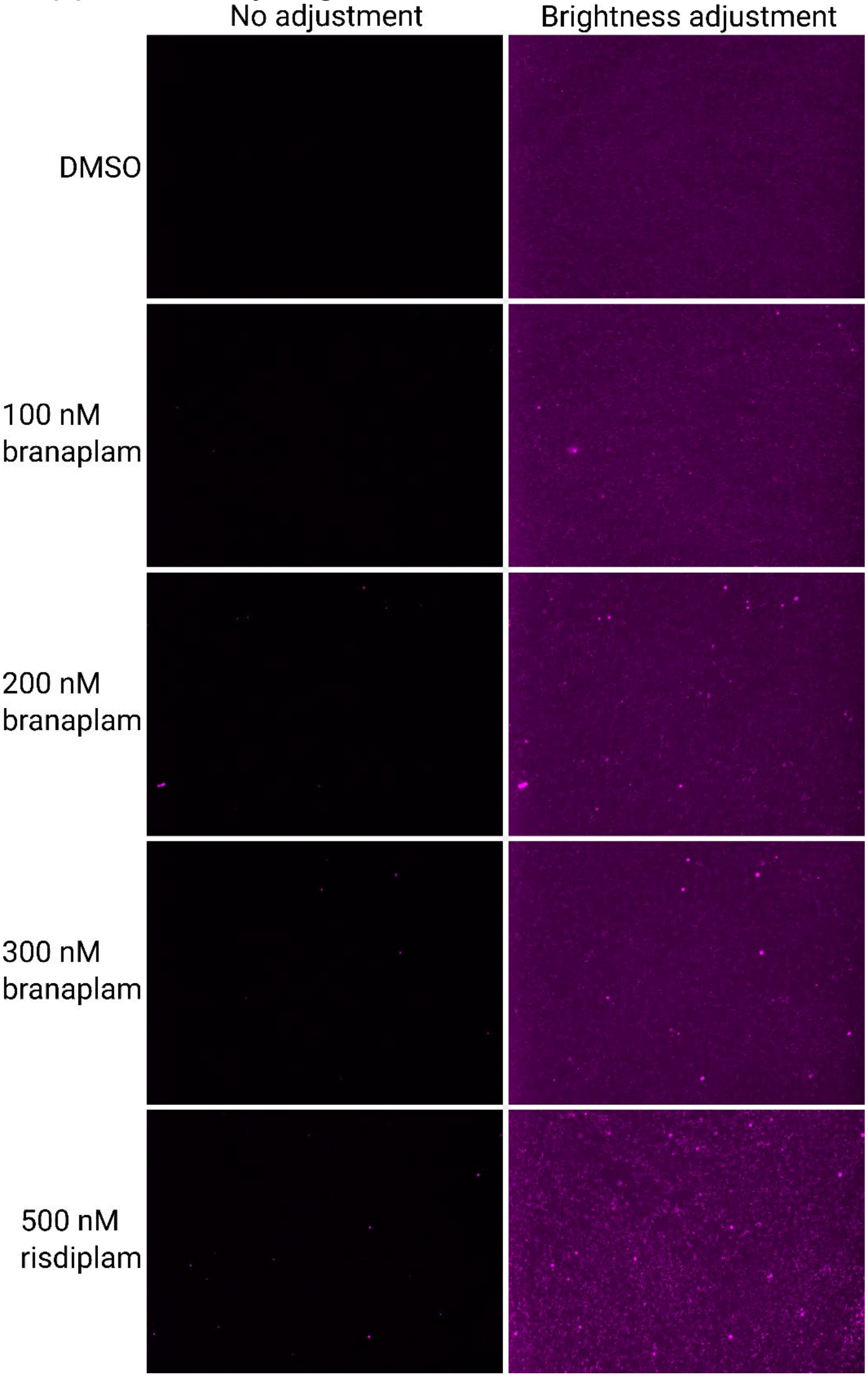

Supplementary Figure 6. The effect of selected branaplam and risdiplam concentrations on cells treated for two weeks at confluency. Images are shown with no adjustment, highlighting the brightly stained dead nuclei, or with a brightness adjustment to highlight the background autofluorescence.

Supplementary Figure 7

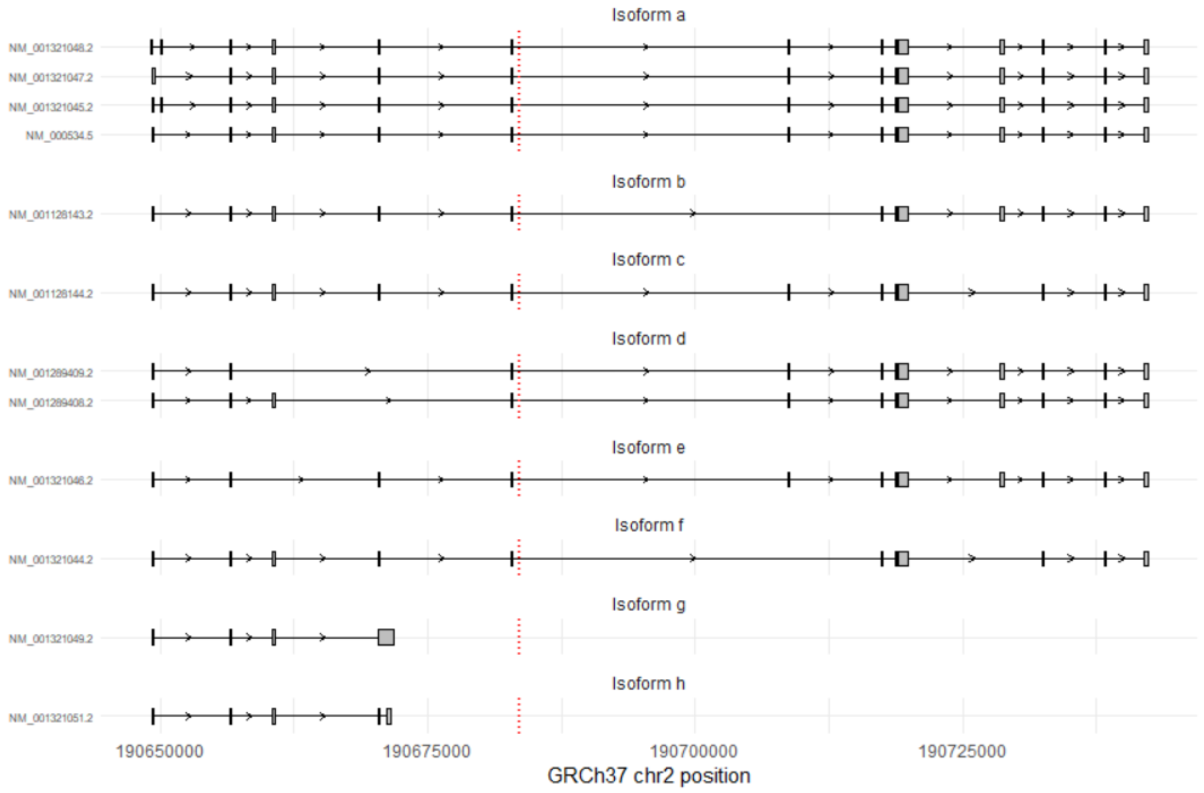

Supplementary Figure 7. *PMS1* alternatively spliced isoforms. NCBI RefSeq curated transcripts for each PMS1 isoform. The pseudoexon location is shown with the dashed red line.

Supplementary Figure 8

a

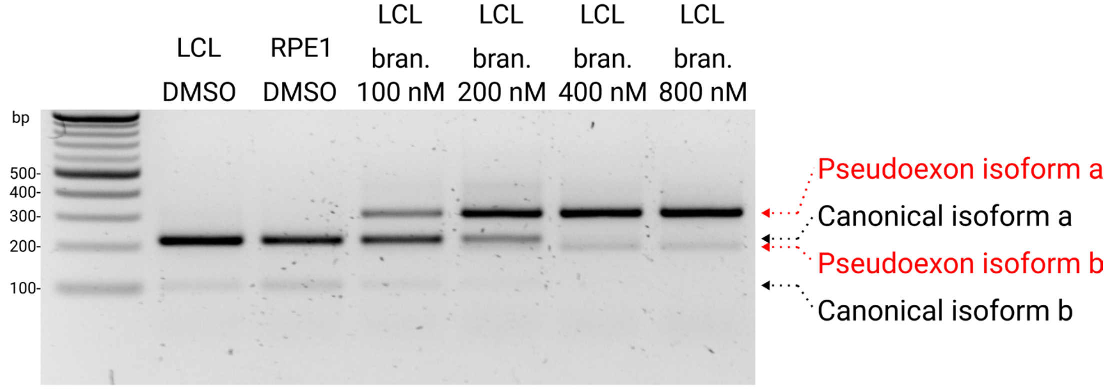

b

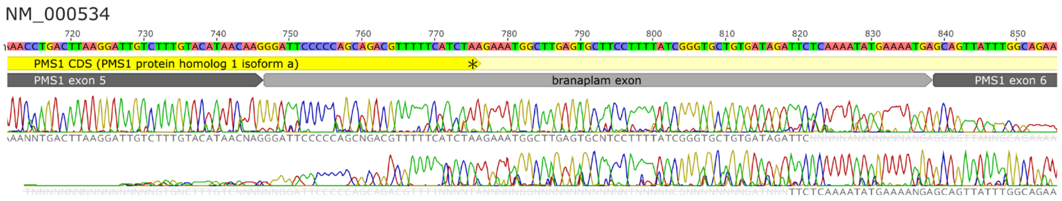

Supplementary Figure 8. *PMS1* pseudoexon. (a) RT-PCR from *PMS1* exon 5-7 showing the two variants, isoform a (includes exon 6) or isoform b (skips exon 6) in LCLs or RPE1 in DMSO control cells and the formation of pseudoexon products (red label) for both isoform a and b across increasing branaplam concentrations in LCLs. The data are from a single experiment. (b) Sanger sequencing traces from an isolated band of pseudoexon inclusion with a PCR from *PMS1* exon 5-6 PCR, with the termination codon indicated by a star. Source data are provided as a Source Data file.

Supplementary Figure 9

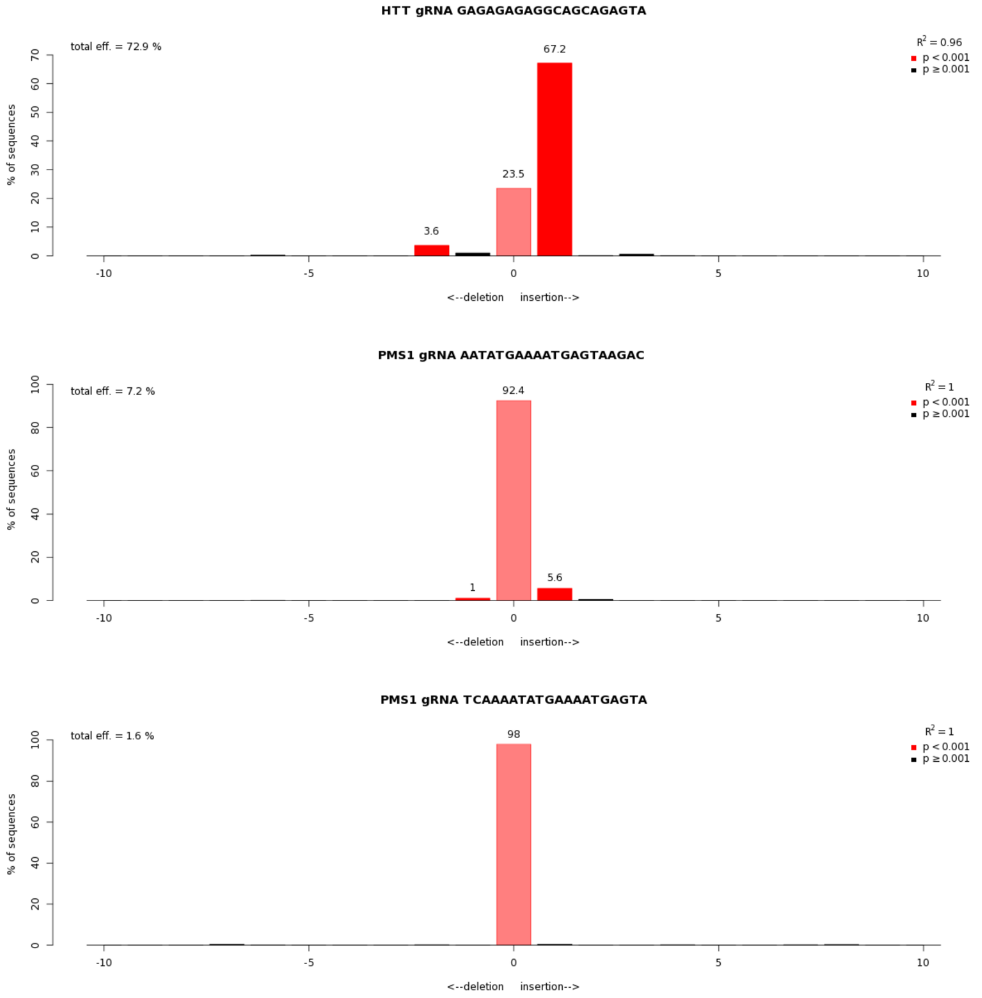

Supplementary Figure 9. Editing outcomes for *HTT* and *PMS1* direct pseudoexon editing by sanger sequencing and quantified by Sanger sequencing trace decomposition. *PMS1* pseudoexon gRNA 1 also targets an intergenic region on chromosome 13, but editing was not quantified.

Supplementary Figure 10

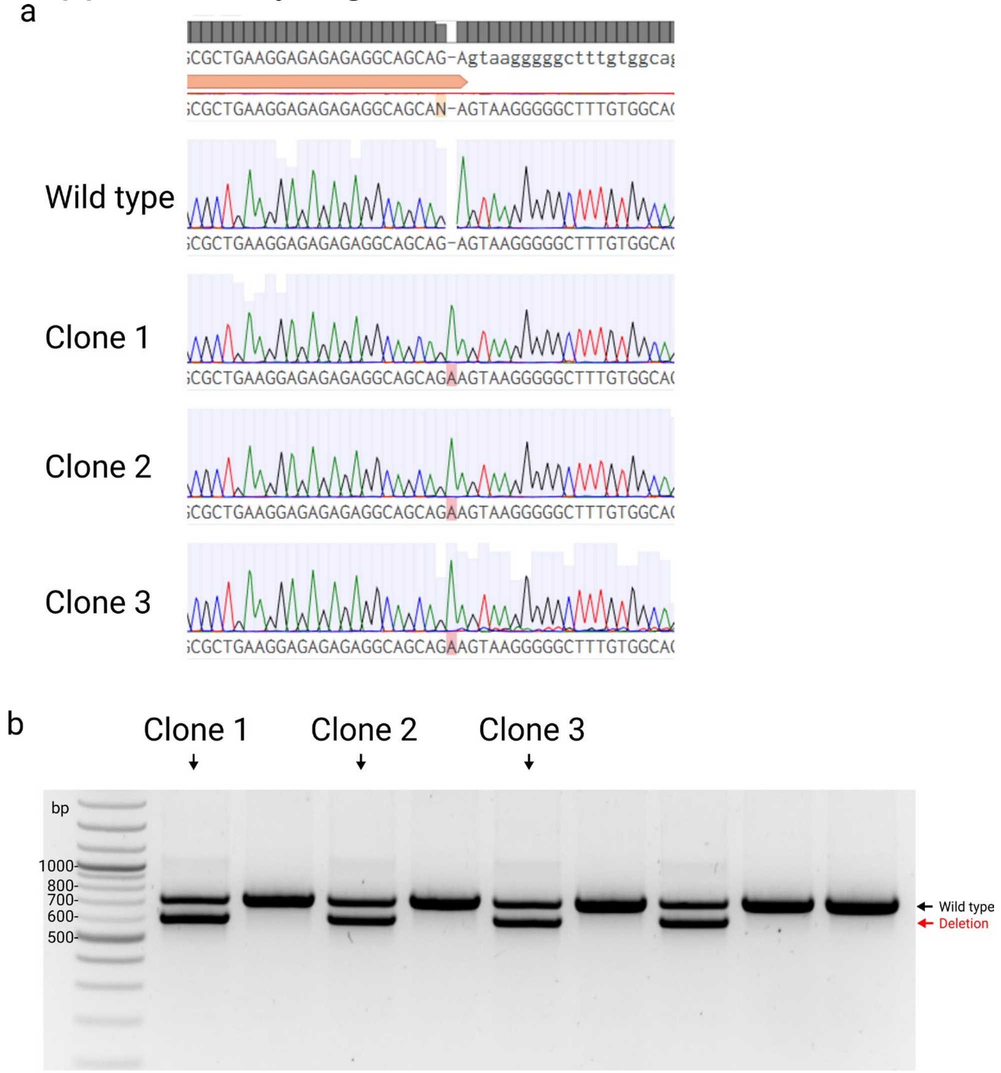

Supplementary Figure 10. Edited cell clones for *HTT* and *PMS1* pseudoexon disruption. (a) Sanger sequencing for three *HTT* edited clones. (b) PCR of *PMS1* genomic region surrounding pseudoexon location of several isolated cell lines. Cell lines heterozygously edited with a deletion of the pseudoexon ("del.") or wild type ("WT") are indicated, and the three clones used for experiments highlighted. The data are from a single experiment.

Supplementary Figure 11

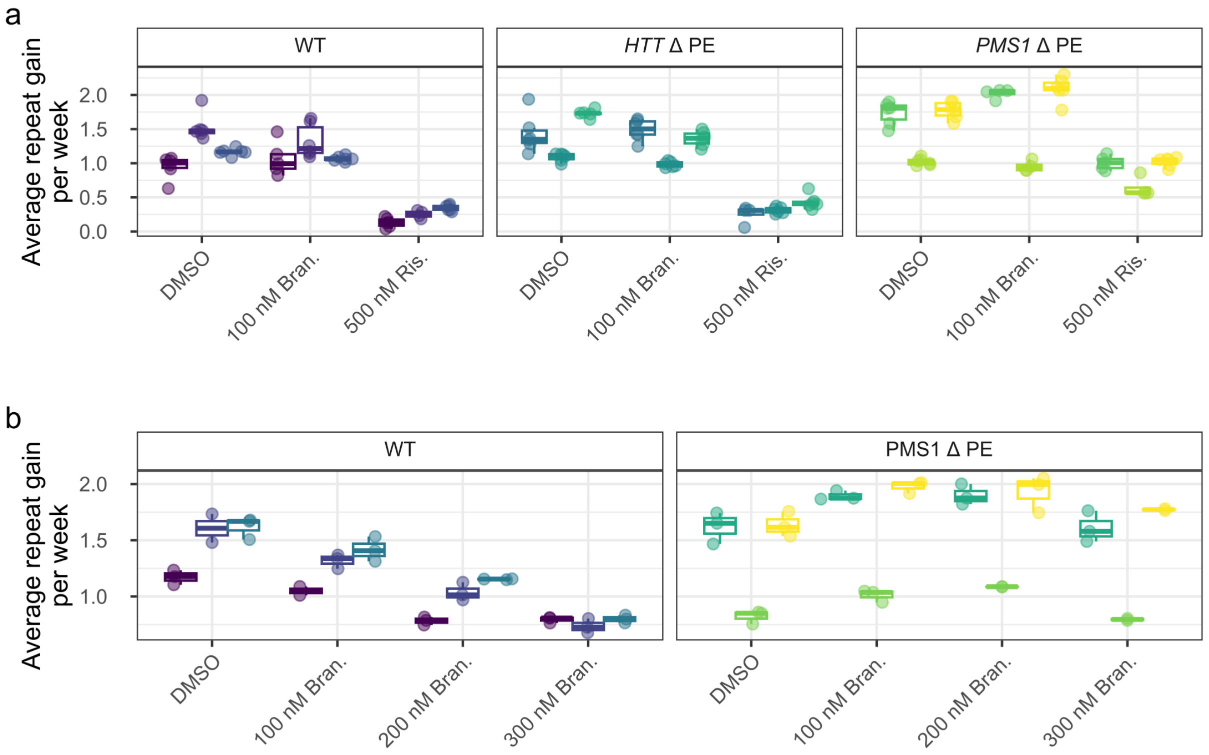

Supplementary Figure 11. Pseudoexon clone phenotypes. The average repeat gain per week after branaplam or risdiplam treatment for the different edited cell lines (dot color). (a) and (b) are equivalent to Figure 7d and Figure 7e without DMSO normalization, respectively. Box plots show the 25th and 75th percentiles (box), median (horizontal line), and range (whiskers, which are capped at 1.5x the inter-quartile range). Source data are provided as a Source Data file.

Supplementary Figure 12

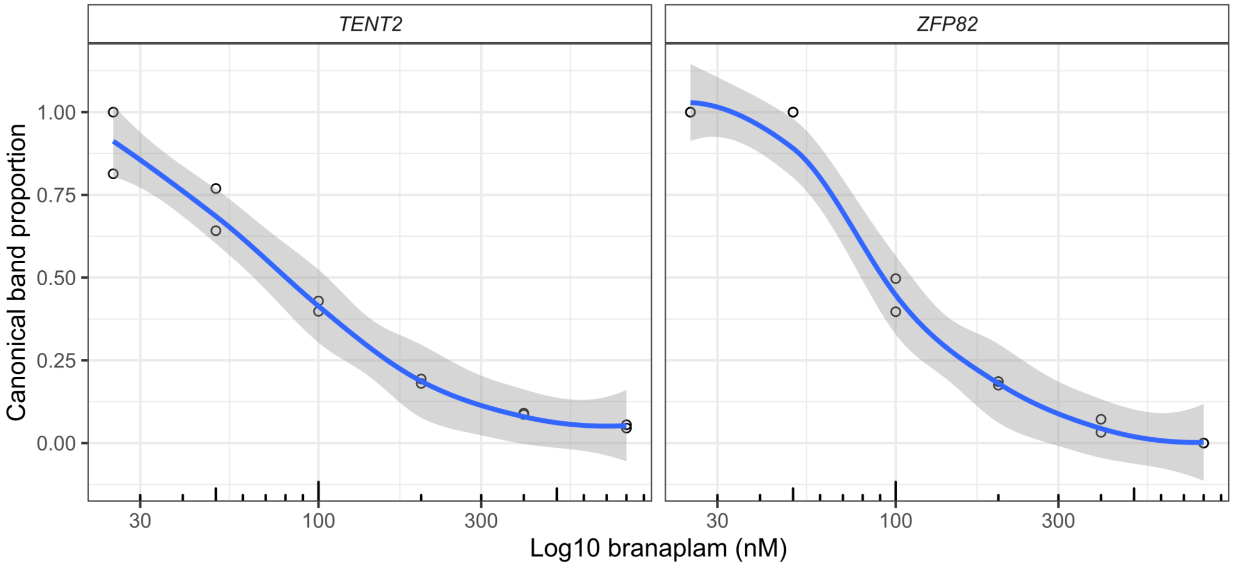

Supplementary Figure 12. Branaplam dose response for *TENT2* (left) and *ZFP82* (right) for canonical splice product after branaplam treatment. Source data are provided as a Source Data file.

Supplementary Figure 13

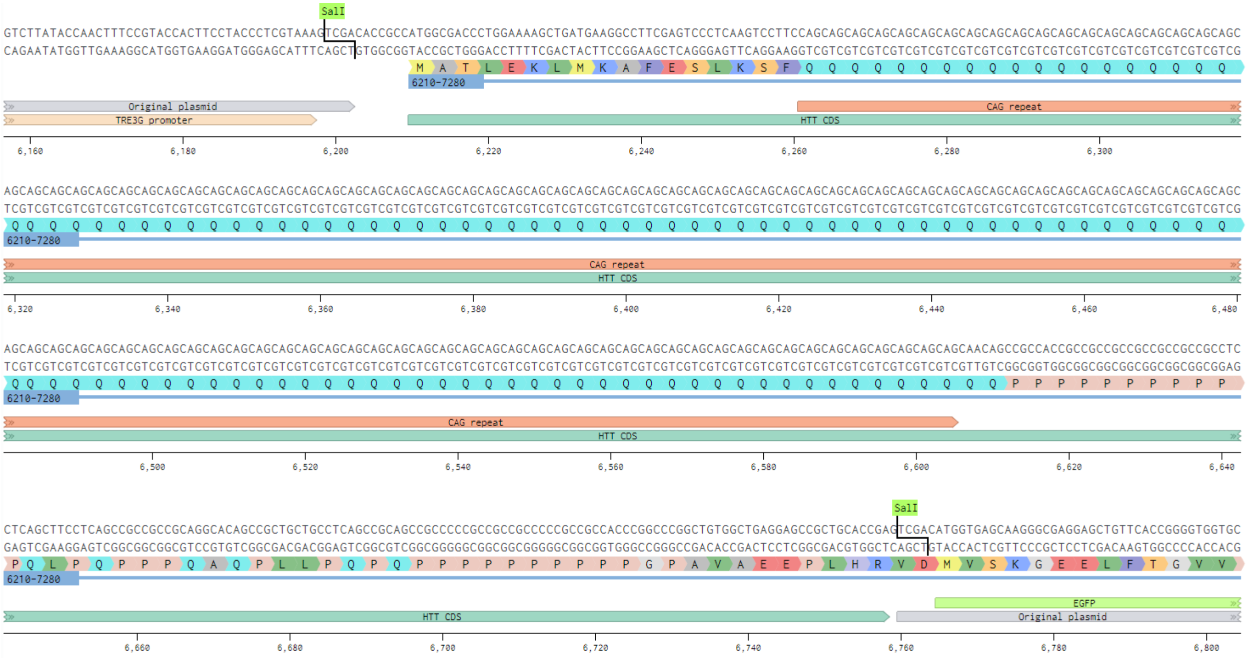

Supplementary Figure 13. AAVS1-CAG115 plasmid. The AAVS1-TRE3G-EGFP (Addgene plasmid # 52343) plasmid was modified to insert the *HTT* exon 1 coding sequence using *Sal*I restriction sites. The *HTT* exon 1 fragment had an expanded CAG repeat tract with 115 units and was inserted to make a GFP fusion protein using the *Sal*I site as a linker sequence. Annotations of the translation and key motifs are under the appropriate sequences, with the original Addgene plasmid # 52343 sequences indicated in gray.

Supplementary Table 1: primers

Name	sequence
AAVS1 5' homology arm F	CTGCCGTCTCTCTCCTGAGT
AAVS1 5' homology arm R	GTGGGCTTGTACTCGGTCAT
CAG repeat sizing 1st PCR F (CAG1)	ATGAAGGCCTTCGAGTCCCTCAAGTCCTTC
CAG repeat sizing 2nd PCR F (CAG1)	/56-FAM/ATGAAGGCCTTCGAGTCCCTCAAGTCCTTC
CAG repeat sizing 1st PCR R (EGFP R)	GTCCAGCTCGACCAGGATG
CAG repeat sizing 2nd PCR R (Hu3)	GGCGGCTGAGGAAGCTGAGGA
EGFP F	CTGCTGCCCGACAACCAC
EGFP probe	/56-FAM/ACCTGAGCA/ZEN/CCCAGTCCGCCCT/3IABkFQ/
EGFP R	TGTGATCGCGCTTCTCGTT
FAN1 KO NGS F	AGTGGTTGAAAAACGTGAGGCA
FAN1 KO NGS R	CCATTGCAGCTTGACCCCTGCT
HTT exon 1 cloning primers with SalI F	CATGTACGgtcgacaccgccATGGCGACCCTGGAAAAGCTG
HTT exon 1 cloning primers with SalI R	CATGTACGgtcgacTCGGTGCAGCGGCTCCTC
HTT exon 49 cloning F	ACCATGGGGGATGCTGCACTGTATCAG
HTT exon 49 cloning R	GGCCTCCAGGATGAAGTG
HTT exon 49 F	GTCTCCAAACTGCCCAGTCAT
HTT exon 49-50 probe	/56-FAM/AACCCTTGAG/ZEN/GCCCTGTCCT/3IABkFQ/
HTT exon 50 R	ACAAACTCTGTGGAGGAGACC
Minigene SDM -1G>A F	GGCAACCCTTGAAgtaagaggcagctcgggag
Minigene SDM -1G>A R	gctgcctcttacTTCAAGGGTTGCCACCAC
Minigene SDM -1G>C F	GGCAACCCTTGACgtaagaggcagctcgggag
Minigene SDM -1G>C R	gctgcctcttacGTCAAGGGTTGCCACCAC
Minigene SDM -1G>T F	GGCAACCCTTGATgtaagaggcagctcgggag
Minigene SDM -1G>T R	gctgcctcttacATCAAGGGTTGCCACCAC
MSH3 KO NGS F	ATGCCCGGCTTGATGCTGTATCG
MSH3 KO NGS R	AGTGTCCTCAAGCTGAAGAACACTGT
PMS1 exon 5-6 probe	/56-FAM/TG TAC ATA A/ZEN/C AAG GCA GTT ATT TGG CAG A/3IABkFQ/
PMS1 exon5 F	TCTCCTCATGAGCTTTGGTATCC
PMS1 exon6 R	ACAGCAGTCCCCAGAACTGA
PMS1 exon7 R	TGGTCTGCATCACACTTTGGA
PMS1 int 5 F	AGGCACCACCTATGAACCAG
PMS1 int 5 R	AGCAACAAGGATGGAAATGG
PMS1 KO NGS F	GCTGAGGATGAATGCAAAATATAGGA
PMS1 KO NGS R	TCAGAGTGGTACTGAAAGGATTCCA
PPP1R12C exon 1 F	TCCAGCCCTCGTTGTCTG
PPP1R12C exon 2 R	GTGCTGGACTCCACCAAC
PPP1R12C rs34521018_A probe	/5SUN/CCTGGTTCA/ZEN/CAGTGGCACCCTGCTCC/3IABkFQ/
PPP1R12C rs34521018_G probe	/56-FAM/CCTGGTTCA/ZEN/CAGTGGCGCCCTGC/3IABkFQ/
PuroR F	GAGTACAAGCCCACGGTG
PuroR probe	/56-FAM/TCGACGGTG/ZEN/TGGCGCGTGGC/3IABkFQ/
PuroR R	TGAGGAAGAGTTCTTGCAGC
SDHA F	TTTGATGCAGTGGTGGTAGG
SDHA probe	/56-FAM/AG CCT AAG A/ZEN/T GAG AGT TCA AGT TGA GTT TGG /3IABkFQ/
SDHA R	CAGAGCAGCATTGATTCCTC

Supplementary Table 2: sgRNAs

Target	sgRNA seq	PAM
FAN1	TGCATGGAGTAACATCCAAG	NGG
MSH3	AAATTGCCCGACATAGAGAG	NGG
PMS1	CAGAGTATCAGATCACAAGA	NGG
HTT pseudoexon 1	GAGAGAGAGGCAGCAGAGTA	NGG
PMS1 pseudoexon 1	AATATGAAAATGAGTAAGAC	NGG
PMS1 pseudoexon 2	TCAAAATATGAAAATGAGTA	NGA
PMS1 pseudoexon flanking LHS	TTAGATGAAAAACGTCTGCT	NGG
PMS1 pseudoexon flanking RHS	CAAAGAATCAACAGTGTAGA	NGG
