# Supplementary material for: Splice modulators target PMS1 to reduce somatic expansion of the Huntington’s disease-associated CAG repeat
Source: Nat Commun. 2024 Apr 12;15:3182. doi: 10.1038/s41467-024-47485-0 (PMC11015039; doi:10.1038/s41467-024-47485-0)
Supplement: Supplementary file 3 — Description of Additional Supplementary Information [file 41467_2024_47485_MOESM3_ESM.pdf]

File Name: NCOMMS-23-33770A_Source_Data.xlsx

Description: Source Data file

File Name: NCOMMS-23-33770A_Supplementary_Information.pdf

Description: Supplementary figures and tables.

File Name: Supplementary_Data_1.xlsx

Description: Branaplam-responsive exons from Monteys et al., 2021 (Extended Data Table 1 & 2); Bhattacharyya et al. (Supplementary Data 2, HTT-C2), 2021; Keller et al., 2022 (Supplementary Data Table 2 & 3); and Ottesen et al., 2023 (Supplementary Table S5, S6, S7). A combined table of each drug responsive exon, the gene, the type (pseudoexon versus existing annotated exon) and the GRCh37/hg19 coordinates.

File Name: Supplementary_Data_2.xlsx

Description: SpliceAI predictions for the effect of variants on the splicing of branaplam responsive exons. The variant coordinates are GRCh37/hg19 position.
