# Supplementary material for: Splice modulators target PMS1 to reduce somatic expansion of the Huntington’s disease-associated CAG repeat
Source: Nat Commun. 2024 Apr 12;15:3182. doi: 10.1038/s41467-024-47485-0 (PMC11015039; doi:10.1038/s41467-024-47485-0)
Supplement: Supplementary file 6 — Reporting Summary [file 41467_2024_47485_MOESM6_ESM.pdf]

nature portfolio

Corresponding author(s): James Gusella

Last updated by author(s): Mar 21, 2024

Reporting Summary

Nature Portfolio wishes to improve the reproducibility of the work that we publish. This form provides structure for consistency and transparency in reporting. For further information on Nature Portfolio policies, see our Editorial Policies and the Editorial Policy Checklist.

Statistics

For all statistical analyses, confirm that the following items are present in the figure legend, table legend, main text, or Methods section.

n/a	Confirmed	
☐	☒	The exact sample size (n) for each experimental group/condition, given as a discrete number and unit of measurement
☐	☒	A statement on whether measurements were taken from distinct samples or whether the same sample was measured repeatedly
☐	☒	The statistical test(s) used AND whether they are one- or two-sided<br>*Only common tests should be described solely by name; describe more complex techniques in the Methods section.*
☐	☒	A description of all covariates tested
☐	☒	A description of any assumptions or corrections, such as tests of normality and adjustment for multiple comparisons
☐	☒	A full description of the statistical parameters including central tendency (e.g. means) or other basic estimates (e.g. regression coefficient) AND variation (e.g. standard deviation) or associated estimates of uncertainty (e.g. confidence intervals)
☐	☒	For null hypothesis testing, the test statistic (e.g. F, t, r) with confidence intervals, effect sizes, degrees of freedom and P value noted<br>*Give P values as exact values whenever suitable.*
☒	☐	For Bayesian analysis, information on the choice of priors and Markov chain Monte Carlo settings
☒	☐	For hierarchical and complex designs, identification of the appropriate level for tests and full reporting of outcomes
☒	☐	Estimates of effect sizes (e.g. Cohen's d, Pearson's r), indicating how they were calculated

Our web collection on statistics for biologists contains articles on many of the points above.

Software and code

Policy information about availability of computer code

Data collection	Data collection was described in the methods. Image acquisition with Celldiscoverer 7 and Incucyte was using Zeiss Zen Blue (version 3.4) and Incucyte software (version 2021C), respectively.
Data analysis	The code for repeat instability analysis was organized as an R package (https://github.com/zachariahmclean/instability, version 0.1.0, http://doi.org/10.5281/zenodo.10825847). The following software's were used for analysis: CellProfiler Analyst (version 3.0.4), CellProfiler (version 4.2.6), CRISPick (version 2022-02-14), CRISPResso (version 2.2.12), flowAI (R package version 1.28.0) flowCore (R package version 2.10.0), flowTime (R package version 1.22.2), GeneMapper (version 5.0), ImageJ (version 1.53e), Incucyte software (version 2021C), marginaleffects (R package version 0.11.1), Python (version 3.8.13), R (version 4.2.1), scikit-image (python package version 0.19.3), SpliceAI (version 1.3.1), StarDist (version 0.8.5), tidyverse (R package version 2.0.0).

For manuscripts utilizing custom algorithms or software that are central to the research but not yet described in published literature, software must be made available to editors and reviewers. We strongly encourage code deposition in a community repository (e.g. GitHub). See the Nature Portfolio guidelines for submitting code & software for further information.

Data

Policy information about availability of data

All manuscripts must include a data availability statement. This statement should provide the following information, where applicable:

- Accession codes, unique identifiers, or web links for publicly available datasets
- A description of any restrictions on data availability
- For clinical datasets or third party data, please ensure that the statement adheres to our policy

All data generated in this paper are provided in the Source Data file. The SpliceAI data used for predictions and results generated in this study are provided as supplementary data files.

Research involving human participants, their data, or biological material

Policy information about studies with human participants or human data. See also policy information about sex, gender (identity/presentation), and sexual orientation and race, ethnicity and racism.

Reporting on sex and gender	*Use the terms sex (biological attribute) and gender (shaped by social and cultural circumstances) carefully in order to avoid confusing both terms. Indicate if findings apply to only one sex or gender; describe whether sex and gender were considered in study design; whether sex and/or gender was determined based on self-reporting or assigned and methods used. Provide in the source data disaggregated sex and gender data, where this information has been collected, and if consent has been obtained for sharing of individual-level data; provide overall numbers in this Reporting Summary. Please state if this information has not been collected. Report sex- and gender-based analyses where performed, justify reasons for lack of sex- and gender-based analysis.*
Reporting on race, ethnicity, or other socially relevant groupings	*Please specify the socially constructed or socially relevant categorization variable(s) used in your manuscript and explain why they were used. Please note that such variables should not be used as proxies for other socially constructed/relevant variables (for example, race or ethnicity should not be used as a proxy for socioeconomic status). Provide clear definitions of the relevant terms used, how they were provided (by the participants/respondents, the researchers, or third parties), and the method(s) used to classify people into the different categories (e.g. self-report, census or administrative data, social media data, etc.) Please provide details about how you controlled for confounding variables in your analyses.*
Population characteristics	*Describe the covariate-relevant population characteristics of the human research participants (e.g. age, genotypic information, past and current diagnosis and treatment categories). If you filled out the behavioural & social sciences study design questions and have nothing to add here, write "See above."*
Recruitment	*Describe how participants were recruited. Outline any potential self-selection bias or other biases that may be present and how these are likely to impact results.*
Ethics oversight	Human cell lines obtained from past studies of HD individuals were approved for secondary use in this study by the MGB Institutional Review Board

Note that full information on the approval of the study protocol must also be provided in the manuscript.

Field-specific reporting

Please select the one below that is the best fit for your research. If you are not sure, read the appropriate sections before making your selection.

☒ Life sciences ☐ Behavioural & social sciences ☐ Ecological, evolutionary & environmental sciences

For a reference copy of the document with all sections, see nature.com/documents/nr-reporting-summary-flat.pdf

Life sciences study design

All studies must disclose on these points even when the disclosure is negative.

Sample size	Sample sizes for each experiment were indicated either in the figure or figure legend. No statistical methods were used to determine sample size. Rather, they were determined using past experience for similar experimental methods.
Data exclusions	Data were excluded for the SpliceAI analysis from Ottesen et al. for intron retention since those splicing events were not determined in the other datasets.
Replication	Technical/biological replicates indicated in the figure or figure legend.
Randomization	The experiments were not randomized since that would not have made sense in any of our studies which were controlled by the same cell line receiving different treatments.
Blinding	Investigators were not blinded for practical project managment and we determined it unnecessary since none of our data were subjectively

Blinding	gathered.

Reporting for specific materials, systems and methods

We require information from authors about some types of materials, experimental systems and methods used in many studies. Here, indicate whether each material, system or method listed is relevant to your study. If you are not sure if a list item applies to your research, read the appropriate section before selecting a response.

Materials & experimental systems

n/a	Involved in the study
☐	☒ Antibodies
☐	☒ Eukaryotic cell lines
☒	☐ Palaeontology and archaeology
☒	☐ Animals and other organisms
☒	☐ Clinical data
☒	☐ Dual use research of concern
☒	☐ Plants

Methods

n/a	Involved in the study
☒	☐ ChIP-seq
☐	☒ Flow cytometry
☒	☐ MRI-based neuroimaging

Antibodies

Antibodies used	PMS1 (68413-1-Ig, Proteintech), B-actin (4967S, Cell Signaling Technology), horseradish peroxidase-linked secondary (NA931 and NA934, GE Healthcare)
Validation	The beta-actin (human) and secondary antibodies are widely used and have been validated by Cell Signaling Technology and GE Healthcare. The PMS1 antibody was validated in this study with western blotting of human PMS1-/- vs PMS1+/+ cells (Supplementary Figure 4c).

Eukaryotic cell lines

Policy information about cell lines and Sex and Gender in Research

Cell line source(s)	LCLs were derived from past studies in our lab at MGH as described in the methods. The hTERT-RPE-1 line was sourced from ATCC (CRL-4000).
Authentication	LCLs were authenticated by microarray genotyping, hTERT-RPE1 was not authenticated.
Mycoplasma contamination	The cell lines were not tested for mycoplasma contamination.
Commonly misidentified lines (See ICLAC register)	No commonly misidentified cell lines were used in the study.

Plants

Seed stocks	*Report on the source of all seed stocks or other plant material used. If applicable, state the seed stock centre and catalogue number. If plant specimens were collected from the field, describe the collection location, date and sampling procedures.*
Novel plant genotypes	*Describe the methods by which all novel plant genotypes were produced. This includes those generated by transgenic approaches, gene editing, chemical/radiation-based mutagenesis and hybridization. For transgenic lines, describe the transformation method, the number of independent lines analyzed and the generation upon which experiments were performed. For gene-edited lines, describe the editor used, the endogenous sequence targeted for editing, the targeting guide RNA sequence (if applicable) and how the editor was applied.*
Authentication	*Describe any authentication procedures for each seed stock used or novel genotype generated. Describe any experiments used to assess the effect of a mutation and, where applicable, how potential secondary effects (e.g. second site T-DNA insertions, mosiacism, off-target gene editing) were examined.*

Flow Cytometry

Plots

Confirm that:

☒ The axis labels state the marker and fluorochrome used (e.g. CD4-FITC).

☒ The axis scales are clearly visible. Include numbers along axes only for bottom left plot of group (a 'group' is an analysis of identical markers).

☒ All plots are contour plots with outliers or pseudocolor plots.

☒ A numerical value for number of cells or percentage (with statistics) is provided.

Methodology

Sample preparation	Described in methods
Instrument	Described in methods
Software	Described in methods
Cell population abundance	Described in figure
Gating strategy	FlowAI was used to QC the data and standard forward and side scatter gates were set.

☐ Tick this box to confirm that a figure exemplifying the gating strategy is provided in the Supplementary Information.
